# Dyslipidemia: prevalence and association with precancerous and cancerous lesions of the cervix; a pilot study

**DOI:** 10.1186/s12944-023-01997-8

**Published:** 2024-01-06

**Authors:** Gakii Fridah Mwangi, Nixon Niyonzima, Raymond Atwine, Deusdedit Tusubira, Godfrey R Mugyenyi, Frank Ssedyabane

**Affiliations:** 1 Department of Medical Laboratory Science, Mbarara University of Science and Technology (MUST), P.O. Box 1410, Mbarara, Uganda; 2 Department of Pathology, Mbarara University of Science and Technology (MUST), P.O. Box 1410, Mbarara, Uganda; 3 Uganda Cancer Institute (UCI), P.O. Box 3935, Kampala, Uganda; 4 Department of Obstetrics and Gynecology, Mbarara University of Science and Technology (MUST), P.O. Box 1410, Mbarara, Uganda; 5 Department of Biochemistry, Mbarara University of Science and Technology (MUST), P.O. Box 1410, Mbarara, Uganda

**Keywords:** Dyslipidemia, Cancerous lesions, Precancerous lesions, Cervix

## Abstract

**Background:**

In Sub-Saharan Africa, the prevalence of dyslipidemia is on the rise, with studies showing dyslipidemia as a contributing factor to the progression of premalignant lesions to cervical cancer. In Uganda, cervical cancer and dyslipidemia are common health concerns, considering the increasing trends of dyslipidemia in the general population and inadequate information regarding dyslipidemia and cervical lesions. This study aimed to determine the prevalence of dyslipidemia and its association with precancerous and cancerous lesions of the cervix among women attending a cervical cancer clinic at the Uganda Cancer Institute.

**Methods:**

This cross-sectional study was conducted from February to April 2022 among women with premalignant and malignant lesions of the cervix. Data on social demographics and health-seeking behaviours were collected using a pretested structured questionnaire after written informed consent had been obtained. Pap smear collection preceded visual inspection with acetic acid; cervical biopsies were collected appropriately from eligible participants; and cervical lesions were classified using the Bethesda system 2014. Serum lipids, total cholesterol (T.C.), high-density lipoprotein (HDLc), low-density lipoprotein (LDLc), and triglycerides (T.G.s) were analysed using the COBAS™ 6000 Clinical Chemistry Analyser. The associations were assessed using the chi-square test, and *P* ≤ 0.05 was considered statistically significant.

**Results:**

The overall prevalence of dyslipidemia among women with cervical lesions was 118/159 (74%), and low HDLc was the most prevalent at 64.6% (95% CI 39.0–54.3). High T.C. (*P* = 0.05), high T.G.s (*P* = 0.011), and low HDL-c (*P* = 0.05) showed a significant association with precancerous lesions. High LDL-c (*P* = 0.019), high T.G.s (*P* = 0.02), and high T.G.s (*P* < 0.001) showed a statistically significant association with cancerous lesions.

**Conclusion:**

The prevalence of dyslipidemia was high, with high TC, T.G.s, and low HDL-c significantly associated with precancerous lesions. Also, elevated T.G.s and high LDLc were significantly associated with cancerous lesions. Women may benefit from dyslipidemia screening along with cervical cancer screening.

**What this study adds:**

The present study builds upon previous findings suggesting a link between dyslipidemia and cervical lesions by investigating the relationship between these two factors, specifically in women of this geographical location, where we need adequate information on these associations.

## Background

Cervical cancer is among the most common cancers in women worldwide. As of 2018, approximately 570,000 women were diagnosed with cervical cancer globally, and 311,000 women died as a result [1]. The incidence and mortality rates of cervical cancer in Sub-Saharan Africa (S.S.A.) are higher than those in any other region of the world, with the age-standardized incidence rate (A.S.R.) of cervical cancer in Southern Africa being the highest in the world, at 43.1 per 100,000 [2]. In Uganda, cancer of the cervix is the most prevalent form of cancer among women aged 15 to 44 [3]. Approximately 3.6% of women in the general population have cervical human papillomavirus (HPV) 16/18 infection, yet HPVs 16 or 18 are responsible for 57.0% of invasive cervical cancers [3].

Precancerous lesions occur when cervical tissue undergoes dysplasia and can be identified by Pap tests or visual inspection with acetic acid. Early treatment lowers the risk of developing cervical cancer [4]. One to five percent of adult females in the general population have cervical dysplasia, primarily in women between the ages of 25 and 35 [5]. If left unchecked, abnormal cells eventually develop into cervical cancer [6]. The primary cause of cervical cancer is HPV infection, which often arises in adolescence following the first sexual encounter [7]. Other risk factors include sexual debut at ≤ 18 years old, many sexual partners or one partner who is high risk (many sexual partners) [8], smoking [9, 10], long-term use of oral contraceptives [10], immunosuppression [11], multiparity [12], and other sexually transmitted diseases such as Chlamydia infection [13] and Human immunodeficiency virus (H.I.V.) infection [11].

The imbalance of lipids, including triglycerides (T.G.s), high-density lipoprotein (HDL-c), low-density lipoprotein cholesterol (LDL-c), and total cholesterol (T.C.), is known as dyslipidemia. This disorder, which can cause cardiovascular disease with serious complications, arises from diet, tobacco use, or genetics. According to Pappan and Rehman [14], it has been linked to the progression of cervical cancer [15]. Even though dyslipidemia has generally been viewed as a disease of wealth, it is becoming increasingly apparent that dyslipidemia is equally frequent in low-income locations such as sub-Saharan Africa [16]. A substantial percentage of women (34.3%, according to research done in South Africa, aged 18–90 suffered from dyslipidemia [17]. Numerous research studies have demonstrated the link between dyslipidemia and chronic diseases like cardiovascular disease (CVD) and type 2 diabetes [18]. However, various studies have produced contradictory findings, and it is still disputed if and to what extent T.G., TC, HDL-c, and LDL-c promote the growth of cervical lesions.

There was an association between dyslipidemia and the presence of high-grade squamous intraepithelial (HSIL), as reported by Frontela-Noda, Delgado-Herrera [19]. Furthermore, a cross-sectional study evaluating the association between serum cholesterol level and abdominal obesity in men and women from Iran aged 25–65 years by Veghari, Sedaghat [20] reported an association between central adiposity and elevated serum cholesterol. An inverse relationship has been reported between blood cholesterol levels and cancer risk [15]. Evidence from preclinical studies shows that dyslipidemia increases the risk of development and growth of cancer by modifying multiple additive risk pathways [21]. A study performed in Korea concluded that increased serum triglycerides predict an adverse prognosis for cervical cancer [22]. In their case–control study in India, Kushwah, Mishra [23] reported significantly abnormal levels of TG, HDL-c, and LDL-c in cervical cancer patients and concluded that dyslipidemia could be a risk factor.

Cases of premalignant lesions and cancer of the cervix are still high in Uganda, and there is an increase in the prevalence of dyslipidemia in Sub-Saharan Africa. While high-risk HPV 16 and 18 infections are the main risk factors for cervical cancer, dyslipidemia has been linked to the progression of cervical lesions, and each of these studies has shown variations in their findings depending on the study population. It is crucial to understand the association between dyslipidemia and cervical lesions among women, no such studies have been carried out in Uganda, whose population is increasingly adopting a sedentary lifestyle and hence more susceptible to lipid disorders. Therefore, this study investigated the prevalence and association between dyslipidemia and cervical precancerous and cancerous lesions at a cancer referral and care facility in Uganda.

## Methods

### Study design and settings

This was a cross-sectional study between February and April 2022 to assess the prevalence and association between dyslipidemia and cervical lesions among women at the cervical cancer clinic of the Uganda Cancer Institute (U.C.I.) Mulago in Kampala, Central Region Uganda, is the country’s only national cancer referral hospital. U.C.I. has three pathologists, 18 laboratory technologists, and four histotechnicians who can process 158 histology and 103 cytology samples monthly.

### Inclusion and exclusion criteria

This study included women between the ages of 21 and 50 attending the cervical cancer clinic of U.C.I. who provided written informed consent. The study excluded women above 50 years to control for age as a confounder for dyslipidemia.

### Data collection

The study team collected data on social demographics (age and marital status [single, married, divorced, or windowed]) and risk factors/health-seeking behaviours (use of family planning method, age at first sexual contact, number of sexual partners, and number of visits for routine screening) from each participant using a validated interviewer-administered questionnaire.

### Study/laboratory procedures

#### Collection of cervical specimens

Cervical specimens were collected by the clinic nurses, whereby a sterile speculum was used to access the cervix, and samples from the endo and ectocervix were collected and transferred to a clean, well-labelled glass slide. The smears were fixed immediately with 95% ethyl alcohol for 15 min and stained using the Papanicolaou staining technique [24]. The specimens were analyzed for adequacy and then reported and classified using the Bethesda system 2014 [25].

#### Collection of biopsies

Women with atypical squamous cells cannot exclude HSIL (ASC-H), HSIL, or suspicious cancer results during screening had a cone/punch biopsy sample obtained for histological examination. Samples were fixed in 10% formaldehyde for 48 h and then processed as described by Dey [24] in a closed system automatic tissue processor for 14 h using the Slee™ tissue processor (SLEE medical GmbH Nieder-Olm, Germany). The tissues were embedded in paraffin wax, chilled, sectioned at a 3–5 µm (μm) thickness, and stained with the hematoxylin and eosin (H & E) staining procedure [24]. The outcomes of biopsies were reported as cervical intraepithelial neoplasia (C.I.N.)1, CIN2, CIN3/carcinoma in situ (C.I.S.), or invasive cervical cancer.

### Processing of blood samples for lipid profile

The study team obtained non-fasting blood samples from the antecubital vein under aseptic conditions after cervical cancer screening. The blood sample was allowed to clot and centrifuged at 3000 rotations per minute (rpm) for 5 min, and the acquired serum was separated into serum separator tubes using a pipette. The samples were loaded into the COBAS™ 6000 Clinical Chemistry Analyser (Roche Diagnostics International AG Rotkreuz, Switzerland) and were analysed according to Roche’s recommendations.

### Quality assurance and control

For cervical specimens, adequacy was guaranteed by ensuring that endo and ectocervix were sampled and all the stains were prepared and stored according to the protocols for the pap stain. The reagents were of the right amount, filtered and at the correct potential of hydrogen (pH).

Experienced pathologists and cytotechnologists examined the slides, and every tenth slide was picked and evaluated by a second pathologist for quality control.

For the lipid profile, commercially available control materials, continuous flow analysis (C.F.A.), and water were used as calibrators for photometric measurements: I.S.E. (ion-selective electrode) standard low (S1), I.S.E. standard high (S2), and I.S.E. standard high (S3) compensators were used as calibrators. Precicontrol clinchem1 (PCCC1) and PCCC2 were run daily as controls to ensure quality results.

The results were analysed daily and periodically while plotting and monitoring the Levy-Jennings chart. These results were interpreted using European Society of Cardiology criteria for dyslipidemia: T.C. above 200 mg/dL, LDL-c above 116 mg/dL, and T.G. levels of more than 150 mg/dL or decreased HDL-c below 35 mg/dL [26].

### Statistical methods and data analysis

The study enrolled 216 participants; 13 were excluded from analysis due to unsatisfactory Pap smears**.** Data from 203 participants were entered into Epidata Analysis (Classic) v2.2.3 software (Jens Lauritsen Odense M, Denmark, Europe) for analysis.

Data on social demographics, health-seeking behaviors, and exposures were presented in the form of frequencies and percentages, and the prevalence was presented as a percentage using a bar graph. The chi-square test was used to determine the associations between dyslipidemia and precancerous and cancerous lesions, where a *P*-value of ≤ 0.05 was considered statistically significant.

## Results

### Demographic characteristics of study participants

The mean age of the study participants was 39 ± 6 years. The majority (70%) used family planning, 74% reported having no more than two sexual partners, and 82% had their first sexual contact before the age of 18 years. The differences between lipid parameters of all groups were statistically significant; T.G.s and LDL-c had a *P*-value of 0.001, while T.C. and HDL-c had a* P*-value of 0.004 and 0.014, respectively. In the normal group, TC/HDL-C was 3 ± 1; in the precancerous group, LDL-C/HDL-C ratio was 2.6 ± 1.7, while the TG/HDL-C ratio among women with cancerous lesions was 6.9 ± 11.7 (Table 1).

**Table 1:** Demographic characteristics of study participants attending the cervical cancer clinic at Uganda Cancer Institute, Mulago-Kampala, between February 2022 and April 2022

**Variable**		**Negative**	**Precancerous lesions**	**Cancerous lesions**	**Test**	***P*-value**
		***N*** **= 44**	***N*** **= 136**	***N*** **= 23**		
		**f(%)**	**f(%)**	**f(%)**		
**Age**		36.61 (7.09)	39.63 (6.87)	40.39 (6.64)	ANOVA	0.028*
**Age group**	20–24	1(2.3%)	0(0.0%)	0(0.0%)	Fisher’s exact	0.023*
	25–29	8(18.2%)	12(8.8%)	1(4.3%)		
	30–34	8(18.2%)	23(16.9%)	2(8.7%)		
	35–39	10(22.7)	27(19.9%)	7(30.4%)		
	40–44	11(25%)	35(25.7%)	7(30.4%)		
	45–50	6(13%)	39(28.7%)	6(26%)		
**Family planning**	No	10 (22.7%)	46 (33.8%)	5 (21.7%)	Chi-square	0.25
	Yes	34 (77.3%)	90 (66.2%)	18 (78.3%)		
**Marital status**	Married	22 (50.0%)	87 (64.0%)	8 (34.8%)	Fisher’s exact	0.055
	Single	9 (20.5%)	23 (16.9%)	4 (17.4%)		
	Divorced	10 (22.7%)	18 (13.2%)	9 (39.1%)		
	Windowed	3 ( 6.8%)	8 ( 5.9%)	2 ( 8.7%)		
**Number of lifetime sexual partners**		1.646 (1.57)	1.74 (1.28)	2.35 (1.67)	ANOVA	0.11
**Age at sexual debut**		16.29 (2.81)	16.34 (2.76)	15.22 (2.54)	ANOVA	0.19
**Total cholesterol (mg/dl)**		155.72 (31.12)	173.37 (47.68)	196.05 (64.56)	ANOVA	0.004*
**LDL-c (mg/dl)**		97.26 (32.05)	103.74 (43.62)	137.27 (56.58)	ANOVA	0.001*
**TG-c (mg/dl)**		92.27 (39.56)	121.58 (84.07)	164.09 (74.51)	ANOVA	0.001*
**HDL-c (mg/dl)**		64.39 (32.06)	49.85 (29.09)	46.49 (33.86)	ANOVA	0.014*
**TC/HDL**		2.97 (1.47)	4.67 (3.85)	6.93 (7.52)	ANOVA	< 0.001*
**LDL/HDL**		1.78 (0.83)	2.62 (1.72)	4.55 (4.37)	ANOVA	< 0.001*
**TG/HDL**		1.92 (1.57)	3.57 (4.41)	6.87 (11.64)	ANOVA	0.002*

Continuous variables, including age, number of lifetime sexual partners, age at sexual debut, and all lipids, were presented as mean (standard deviation)

*LDLc* Low-density lipoprotein, *HDLc* High-density lipoprotein, *T.G.s* triglycerides, *TC* total cholesterol

^*^P-value ≤ 0.05

### Prevalence of dyslipidemia

**Fig. 1:**
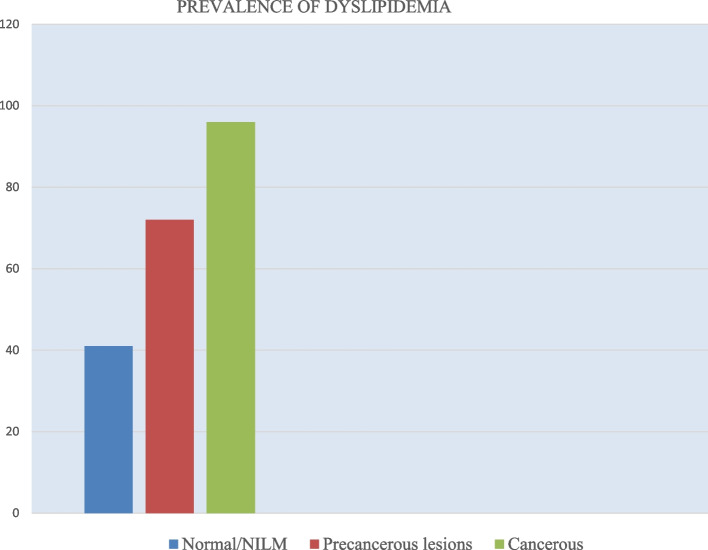
Overall prevalence of dyslipidemia among women in the study attending the cervical cancer clinic at Uganda Cancer Institute, Mulago-Kampala, between Feb 2022 and April 2022. Abbreviation: NILM- Negative for intraepithelial lesion/Malignancy

Dyslipidemia was observed in 74% (118/159) of participants with cervical lesions. For those with precancerous lesions, the prevalence was 73%(96/136), and for cancerous lesions, the prevalence was 96% (22/23). The prevalence among the normal/negative for intraepithelial lesions was 41% (18/44) (Fig. 1). When comparing the prevalence of dyslipidemia between normal, precancerous, and cancerous groups, statistically significant differences were observed. Increased LDL-C had a *P*-value of 0.007; both T.G.s and T.C. had a *P*-value of < 0.001. In contrast, HDL-c had a *P*-value of 0.011 (Table 2).

**Table 2:** The prevalence of dyslipidemia among normal, precancerous, and cancerous lesions of the cervix in women attending the cervical cancer clinic at Uganda Cancer Institute, Mulago-Kampala, between Feb 2022 and April 2022

**Variables**		**Negative**	**Precancerous lesions**	**Cancerous lesions**	**Test**	***P*-value**
		***N*** **= 44**	***N*** **= 136**	***N*** **= 23**		
		**f(%)**	**f(%)**	**f(%)**		
**HDL-c**	HDL-c DYSLIIDEMIA	6 (13.6%)	39 (28.7%)	11 (47.8%)	Chi-square	0.011*
	NORMAL HDL-c	38 (86.4%)	97 (71.3%)	12 (52.2%)		
**TG-c**	NORMAL TRIG	40 (90.9%)	104 (76.5%)	10 (43.5%)	Fisher’s exact	< 0.001*
	TRIG DYSLIPIDEMIA	4 ( 9.1%)	32 (23.5%)	13 (56.5%)		
**LDL-c**	NORMAL LDL-c	34 (77.3%)	91 (66.9%)	9 (39.1%)	Chi-square	0.007*
	LDL-c DYSLIPIDEMIA	10 (22.7%)	45 (33.1%)	14 (60.9%)		
**TC**	NORMAL TCHOL	41 (93.2%)	98 (72.1%)	13 (56.5%)	Fisher’s exact	< 0.001*
	TCHOL DYSLIPIDEMIA	3 ( 6.8%)	38 (27.9%)	10 (43.5%)		

*LDLc* Low-density lipoprotein, *HDLc* High-density lipoprotein, *T.G.s* triglycerides, *TC* total cholesterol

^*^P-value ≤ 0.05

The prevalence of low HDLc was 12/27(44%) among women with high-grade lesions and 14/65 (22%) in women with low-grade lesions. The prevalence of more than one type of dyslipidemia was highest among those with cancerous lesions. Precancerous lesions were diagnosed in 136/203 (67%) study participants (Table 3).

**Table 3:** Prevalence of dyslipidemia among women with precancerous lesions of the cervix attending the cervical cancer clinic at Uganda Cancer Institute, Mulago-Kampala, between Feb 2022 and April 2022

**Variables**		**ASUS**	**LSIL**	**ASC-H**	**HSIL**	**TEST**	***P*-value**
		***N*** **= 22**	***N*** **= 65**	***N*** **= 22**	***N*** **= 27**		
**HDL-c**	HDL-c DYSLIPIDEMIA	6 (27%)	14 (22%)	7 (32%)	12 (44%)	Chi-square	0.05*
	NORMAL HDL-c	16 (73%)	51 (78%)	15(68%)	15 (56%)		
**TG-c**	NORMAL TRIG	18 (82%)	54 (83%)	17 (77%)	15(52%)	Fisher’s exact	< 0.002*
	TRIG DYSLIPIDEMIA	4 (18%)	11 (17%)	5(23%)	13 (48%)		
**LDL-c**	NORMAL LDL-c	14 (64%)	45 (69%)	14 (64%)	18 (67%)	Chi-square	0.72
	LDL-c DYSLIPIDEMIA	8 (36%)	20 (31%)	8 (36%)	9 (33%)		
**TC**	NORMAL TCHOL	18 (82%)	49 (75%)	14 (64%)	17 (63%)	Fisher’s exact	0.036*
	TCHOL DYSLIPIDEMIA	4 (18%)	16 (25%)	8 (36%)	10 (37%)		

*ASC-H* Atypical squamous cells cannot exclude HSIL, *ASCUS* Atypical squamous cells of undetermined significance, *LSIL* Low-grade squamous intraepithelial lesion, *HSIL* High-grade squamous intraepithelial lesion, *NILM* Negative for intraepithelial lesion/malignancy, *LDLc* Low-density lipoprotein, *HDLc* High-density lipoprotein, *T.G.s* Triglycerides; TC-total cholesterol

^*^P-value ≤ 0.05

Cancerous lesions among the WDSCC group were 15/23 (65%), while moderate-poorly differentiated S.C.C. was 8/23 (35%). The prevalence of high T.G.s was 7/15(46%) in well-differentiated S.C.C. and high LDL-c was 5/8(62.5%) in the moderate-poorly differentiated S.C.C. (Table 4).

**Table 4:** Prevalence of dyslipidemia among women with cancerous lesions of the cervix attending the cervical cancer clinic at Uganda Cancer Institute, Mulago-Kampala, between Feb 2022 and April 2022

**Variables**		**Well-differentiated**	**Moderate-poorly**	**Test**	***P*-value**
		**S.C.C**	**Differentiated S.C.C**		
		***N*** **= 15**	***N*** **= 8**		
**HDL-c**	HDL DYSLIPIDEMIA	7 (46.7%)	4 (50.0%)	Fisher’s exact	0.018*
	NORMAL	8 (53.3%)	4 (50.0%)		
**TG-c**	NORMAL TRIG	8 (53.3%)	2 (25.0%)	Fisher’s exact	< 0.001*
	TRIG DYSLIPIDEMIA	7 (46.7%)	6 (75.0%)		
**LDL-c**	NORMAL LDL-c	6 (40.0%)	3 (37.5%)	Fisher’s exact	0.099
	LDL DYSLIPIDEMIA	9 (60.0%)	5 (62.5%)		
**TC**	NORMAL TCHOL	9 (60.0%)	4 (50.0%)	Fisher’s exact	0.019*
	TCHOL DYSLIPIDEMIA	6 (40.0%)	4 (50.0%)		

*LDLc* low-density lipoprotein, *HDLc* high-density lipoprotein, *T.G.s* triglycerides, *TC* total cholesterol, *SCC* squamous cell carcinoma

^*^P-value ≤ 0.05

### Association between dyslipidemia and precancerous lesions

A significant association was observed between low-grade lesions and high T.C. (*P* = 0.05). High T.G.s and low HDL-c were also significantly associated with high-grade precancerous lesions *P* = 0.011 and 0.05, respectively. (Table 5).

**Table 5:** Association between dyslipidemia and precancerous lesions of the cervix among women attending the cervical cancer clinic at Uganda Cancer Institute, Mulago-Kampala, between Feb 2022 and April 2022

	**Low-grade lesions**				**High-grade lesions**			
	f	Chi^2^value	Df	*P*-value	f	Chi^2^value	Df	*P-*value
↑LDLc	28	1.269	1	0.260	17	0.089	1	0.7652
↑TGs	15	1.565	1	0.2109	18	6.481	1	0.011*
↑T.C	20	3.77	1	0.052*	18	2.942	1	0.0863
↓HDLc	20	1.607	1	0.205	19	0.382	1	0.051*

*LDLc* low-density lipoprotein, *HDLc* high-density lipoprotein, *T.G.s* triglycerides, *TC* total cholesterol, *DF* degrees of freedom

^*^P-value ≤ 0.05

### Association between dyslipidemia and cancerous lesions

This study observed an association between high LDL-c(*P* = 0.019), high T.G.s (*P* = 0.02), and well-differentiated squamous cell carcinoma (WDSCC), while high T.G.s (*P* < 0.001) showed a significant association with moderately/poorly differentiated squamous cell carcinoma. (Table 6).

**Table 6:** Association between dyslipidemia and cancerous lesions of the cervix among women attending the cervical cancer clinic at Uganda Cancer Institute, Mulago-Kampala, between Feb 2022 and April 2022

Variable	Well differentiated S.C.C.				Moderately/poorly differentiated S.C.C			
	f	Chi^2^value	df	*P*-value	f	Chi^2^value	df	*P*-value
↑LDLc	9	5.444	1	0.02*	5	3.597	1	0.057
↑TGs	7	5.403	1	0.02*	6	12.870	1	< 0.001*
↑TC	6	2.073	1	0.15	4	2.947	1	0.08
↓HDLc	7	3.324	1	0.068	4	2.484	1	0.115

*LDLc* Low-density lipoprotein, *HDLc* High-density lipoprotein, *T.G.s* triglycerides, *TC* total cholesterol, *DF* degrees of freedom

^*^P-value ≤ 0.05

## Discussion

### Prevalence of dyslipidemia among women with precancerous and cancerous lesions

In the analysis, 74% of the participants with cervical lesions had dyslipidemia. This is a novel study that examines dyslipidemia, a component of metabolic syndrome (MetS), in terms of prevalence and association with precancerous and cancerous lesions. There are few studies available to make adequate comparisons. However, dyslipidemia has been reported in other malignancies. For instance, in their case–control study, Blanc-Lapierre, Spence [27] found that prostate cancer controls aged 40–59 years and those above 60 had a MetS prevalence of 22% and 34% in Montreal, Canada. Also, in a retrospective study at Peking Medical College (Beijing, China) on the prevalence of obesity, dyslipidemia, and overweight in individuals with intracranial germ cell tumors, 49 (46.2%) and 86 (81.1%) individuals were identified as having dyslipidemia and being overweight or obese, respectively [28].

This study revealed that high LDL-c accounted for 37.1%, followed by low HDL-c at 31% among those with cervical lesions. Similarly, low HDL-c levels and prostate cancer were found to have a strong correlation. There was a 60% greater risk of prostate cancer in people with high LDL-c levels in a hospital-based case–control study in the U.S.A. [29]. These findings concur with a cross-sectional baseline survey study in the Vaal region of South Africa, where they reported low HDL-c as the most common type of dyslipidemia [17]. Considering these studies, there is a high likelihood that dyslipidemia is indeed associated with malignancies including cervical cancer or its premalignant lesions.

High LDL-c was the most prevalent dyslipidemia in women with LSIL, while high T.G.s was common in the HSIL group. Combined dyslipidemia was more prevalent among women with cancerous lesions, particularly in those with well-differentiated S.C.C. and moderately/poorly differentiated S.C.C. High LDL-c had the highest prevalence in women with well-differentiated S.C.C. In contrast, high T.G. was prevalent in women with moderately/poorly differentiated S.C.C. These outcomes are comparable to those reported by Raju, Punnayanapalya [15], in a case–control study among women from India. These results resonate well with the pathophysiology of dyslipidemia in cervical cancer. Dyslipidemia promotes the proliferation of cancer cells and anti-apoptotic capacity by activating reactive oxygen species (R.O.S.) and Deoxyribonucleic acid (D.N.A.) damage [22, 30], which are known processes underlying cervical dysplasia.

### Association between dyslipidemia and precancerous lesions

This study found a significant association between high T.G.s, low HDL-c, and high-grade lesions. This is similar to a Cuban study that reported a significant association between HSIL and low HDL-c [19]. The current investigation found a significant relationship between elevated T.C. levels and low-grade squamous intraepithelial lesions. Veghari, Sedaghat [20] reported that high total cholesterol was significantly associated with central adiposity, especially in women aged 25–35. Frontela-Noda, Delgado-Herrera [19] also observed elevated central adiposity/abdominal obesity in women who had precancerous lesions and therefore, concluded that abdominal obesity had a greater impact on the likelihood of developing cervical cancer.

There are limited data on the association between dyslipidemia and precancerous lesions; however, in their cross-sectional study aimed at differentiating the lipid profile of cervical epithelial cells depending on the severity of the disease, Sitarz, Czamara [31] found high lipid levels in the cells of Polish women who had HSIL and S.C.C. diagnosis. The enhanced expression of the fatty acid synthase (FASN) and acetyl-CoA carboxylase (A.C.C.) enzymes is linked to the phenomena of de novo lipid synthesis in malignant cells [32].

### Association between dyslipidemia and cancerous lesions

This study presents an association between high LDLc and high T.G.s with WDSCC, indicating that women diagnosed with WDSCC are more likely to have elevated LDL-c and T.G.s or vice versa. Similarly, Jiang, Wang [30] observed a link between high LDL-c and cancerous lesions, while Raju, Punnayanapalya [15] observed an increase in LDL-c values as cervical cancer (CC) progressed from stage I to stage IV. There was no significant association between high T.C. or low HDL-c and WDSCC. Likewise, a retrospective clinical study in China to characterize serum lipids in gynecological cancers and benign disease by Sun, Meng [33] reported no significant association between dyslipidemia and cervical cancer compared to uterine leiomyomas. This study reported an association between high T.G.s and moderate/poorly differentiated S.C.C., as did Ahn, Shin [22], who reported an association between MetS (with emphasis on high T.G.s) in a retrospective study among patients with an early cervical cancer diagnosis in Korea. Consequently, in Ulmer, Bjørge [34] research, high T.G.s were confined to S.C.C.

Lipids are vital in invasion, migration, and metastasis, but they also play a significant role in cancer [35]. The entire cellular metabolism is reprogrammed during carcinogenesis to benefit a cell that divides quickly [36]. Activation of the Akt/Pi3K pathway increases the expression of sterol regulatory element-binding proteins (SREBPs), which is strongly related to lipid and carbohydrate metabolism changes [37]. SREBPs stimulate the synthesis of two key enzymes involved in forming fatty acids, A.C.C. and FASN [32]. Malonyl-CoA is produced by A.C.C., while the reaction of fatty acid chain extension is catalyzed by FASN [32]. Consequently, Akt/PI3K activation promotes lipid production and carbohydrate oxidation in cancer cells. Unsaturated fatty acids (S.F.A.s), crucial signal transducers in cancer that promote proliferation and inhibit apoptosis, are also typically linked to cancer [38, 39]. Stearoyl-CoA desaturase (SCD1) activity is correlated with higher lipid levels in neoplastic cells [40]. Saturated fatty acids are changed by this enzyme into monounsaturated fatty acids (MUFAs) [41]. Epigenetic alterations are also linked to altered gene expression in malignancies, particularly in genes involved in cellular metabolism [42]. D.N.A. methylation, an epigenetic modification, plays a crucial role in gene expression [43].

Lin, Zheng [44] postulated that elevated triglycerides and cholesterol levels could cause hypoimmunity and tumor growth, which would then aggravate the clinical results. At the same time, Sitarz, Czamara [31] concluded that the HSIL and S.C.C. groups have the most mitochondrial genome, whereas the LSIL group has the least. In the past, dysplasia and oncogenesis were linked to cervical epithelial cells with higher copies of the mitochondrial genome [45].

### Strengths and limitations

This study was conducted at the highest level of cancer care and referral health facility in Uganda; therefore, the patient population largely represents the majority of the country. This novel study also used internationally acceptable cut off values for categorizing dyslipidemias. However, this was a cross-sectional study, and it was impossible to make adequate comparisons between grades of cervical lesions. For instance, there were no adenocarcinoma cases included in this study. The study was hospital-based, so these conclusions cannot be easily generalized to the entire population.

## Conclusions

The prevalence of dyslipidemia among women with cervical lesions is high. High T.C. is significantly associated with LSIL, while low HDL-c and high T.G. are associated with HSIL. High T.G.s and high LDL are associated with cancerous lesions (S.C.C.). Women diagnosed with cervical lesions would benefit if their lipid parameters are assessed and managed accordingly, as this may improve overall and recurrence-free survival. Prospective molecular studies with larger sample sizes should be conducted to provide a clearer picture of the molecular mechanisms behind the observed associations.

## Data Availability

Raw data were generated at the Uganda Cancer Institute. Derived data supporting the findings of this study are available from the corresponding author, MG, on reasonable request.

## Abbreviations

A.C.C.: Acetyl CoA Carboxylase
ASC-H: Atypical Squamous Cells Cannot exclude High-grade squamous intraepithelial lesions
CC: Cervical Cancer
C.I.N.: Cervical Intraepithelial Neoplasia
C.I.S.: Carcinoma In situ
D.N.A.: Deoxyribonucleic acid
FASN: Fatty acid synthase
HDLc: High-density lipoprotein cholesterol
H.I.V.: Human immunodeficiency virus
HPV: Human Papilloma virus
HSIL: High-grade squamous intraepithelial lesions
LDLc: Low-density lipoprotein cholesterol
LSIL: Low-grade squamous intraepithelial lesions
MUFA: Monounsaturated fatty acid
PCCC: Precicontrol clinicalchem
ROS: Reactive oxygen species
S.C.C.: Squamous cell carcinoma
S.C.D.: Stearoyl CoA desaturase
S.F.A.: Saturated fatty acid
SREBPs: Sterol regulatory element-binding proteins
T.C.: Total cholesterol
T.G.s: Triglycerides
U.C.I.: Uganda Cancer Institute
WDSCC: Well-Differentiated Squamous Cell Carcinoma

## Declarations

**Ethics approval and consent to participate**

The Mbarara University of Science and Technology Research Ethics Committee (MUST-2021–311) and the Uganda National Council for Science and Technology (HS2087ES) reviewed and approved the protocol and informed consent documents. The study team also obtained administrative clearance from the Uganda Cancer Institute (SR.06/22) to conduct the study at the institute and received written informed consent from each participant before participation in the study. This study was conducted following the Helsinki Declaration as revised in 2013, and each participant was given an explanation of the study’s purpose and the benefits and risks associated with participation. For confidentiality, all laboratory specimens, evaluation forms, and reports were identified by coded numbers and secured under lock and key.
